# Longitudinal Profiles of Thyroid Hormone Parameters in Pregnancy and Associations with Preterm Birth

**DOI:** 10.1371/journal.pone.0169542

**Published:** 2017-01-06

**Authors:** Lauren E. Johns, Kelly K. Ferguson, Thomas F. McElrath, Bhramar Mukherjee, Ellen W. Seely, John D. Meeker

**Affiliations:** 1 Department of Environmental Health Sciences, University of Michigan School of Public Health, Ann Arbor, Michigan, United States; 2 Epidemiology Branch, National Institute of Environmental Health Sciences, Research Triangle Park, NC, United States; 3 Division of Maternal and Fetal Medicine, Brigham and Women’s Hospital, Harvard Medical School, Boston, MA, United States; 4 Department of Biostatistics, University of Michigan School of Public Health, Ann Arbor, MI, United States; 5 Division of Endocrinology, Diabetes, and Hypertension, Brigham and Women’s Hospital, Harvard Medical School, Boston, MA, United States; John Hunter Hospital, AUSTRALIA

## Abstract

**Introduction:**

Overt thyroid disease in pregnancy is associated with numerous maternal and neonatal complications including preterm birth. Less is known about the contribution of trimester-specific subclinical alterations in individual thyroid hormones, especially in late gestation, on the risk of preterm birth. Herein, we examined the associations between subclinical changes in maternal thyroid hormone concentrations (TSH, total T3, free and total T4), measured at multiple time points in pregnancy, and the odds of preterm birth in pregnant women without clinical thyroid disease.

**Participants and Methods:**

Data were obtained from pregnant women participating in a nested case-control study of preterm birth within on ongoing birth cohort study at Brigham and Women’s Hospital in Boston, MA (N = 439; 116 cases and 323 controls). We measured thyroid hormones in plasma collected at up to four time points in pregnancy (median = 10, 18, 26, and 35 weeks). We used multivariate logistic regression models stratified by study visit of sample collection to examine associations. To reveal potential biological pathways, we also explored these relationships by obstetric presentation of preterm birth (e.g., spontaneous preterm delivery) that have been previously hypothesized to share common underlying mechanisms.

**Results:**

In samples collected at median 10 and 26 weeks of gestation, we found inverse associations between FT4 and the odds of overall preterm birth (odds ratio [OR] = 0.57, 95% confidence interval (CI) = 0.33, 1.00; and OR = 0.53, 95% CI = 0.34, 0.84, respectively). Positive associations were detected for total T3 at these same time points (OR = 2.52, 95% CI = 1.20, 5.31; and OR = 3.40, 95% CI = 1.56, 7.40, respectively). These effect estimates were stronger for spontaneous preterm birth.

**Conclusions:**

Our results suggest that subclinical alterations in individual maternal thyroid hormones may influence the risk of preterm birth, and the strength of these associations vary by gestational age.

## Introduction

Preterm birth (PTB) is among the most frequent causes of global infant and neonatal mortality [1]. While recent medical advances have improved survival among preterm infants, the long-term health and economic consequences associated with prematurity are substantial [1, 2]. Prevention of PTB is a challenge owing to the complexity of its causes, many of which are poorly understood [3].

Maternal thyroid hormones are crucial for normal fetal growth and development, especially neurodevelopment [4]. This is particularly true in the first trimester when the fetus is entirely dependent on the transplacental passage of maternal thyroid hormones [5, 6]. Maternal thyroid hormones also play a physiological role in early placental development by regulating human trophoblast proliferation and invasion [6–10]. Inadequate trophoblast cell invasion may result in abnormal placentation, which notably is a risk factor for preterm delivery [9, 11].

Research has shown that overt hyper- and hypothyroidism in pregnancy are associated with poor maternal and neonatal outcomes [12–16]. However, data on the consequences of milder forms of maternal thyroid dysfunction on the risk of PTB in particular have been less conclusive. Subclinical hypothyroidism or elevated thyrotropin (TSH) has been associated with preterm delivery in some studies [17–20] but not in others [13, 21]. There has also been suggestive evidence that hypothyroxinemia (normal TSH concentrations with low free thyroxine [FT4]) in early pregnancy may increase the risk of prematurity [19]. Notably, these studies are limited by single biomarker measurements from the first or second trimester.

Currently, there are a lack of data on the effects of trimester-specific subclinical alterations in individual parameters of thyroid function, especially in late gestation, on the risk of PTB. The purpose of this study was to examine the associations between subclinical fluctuations in biochemical markers of thyroid function, measured at up to four time points in pregnancy, and the risk of PTB in a nested case-control study of pregnant women without clinical thyroid disease.

## Materials and Methods

### Study population

Participants were part of a nested case-control study of PTB drawn from a prospective birth cohort (the LifeCodes cohort) of pregnant women recruited early in gestation (<15 weeks) at Brigham and Women’s Hospital in Boston, MA. Additional information regarding recruitment and eligibility criteria are described in detail elsewhere [22, 23]. The nested case-control study includes 130 women who delivered preterm (<37 weeks) and 352 randomly selected controls. We additionally excluded from the study, women who reported pre-existing or gestational thyroid disease/conditions based on answers to medical questionnaires administered at each of the study visits (N = 41; e.g., hyper- or hypothyroidism, Graves’ disease, or thyroid cancer) and those who did not provide plasma samples at any study visit (N = 2). The final study population for the current analyses included 116 cases of PTB and 323 controls. The proportion of women delivering preterm did not significantly differ between women included vs. excluded from the current study (χ^2^ = 0.75, p = 0.39). The study protocols were approved by the ethics review board at Brigham and Women’s Hospital (Partners Health Research Committee) and all study participants gave written informed consent.

Gestational age at individual study visits and at delivery were calculated based on last menstrual period and confirmed by first trimester ultrasound [22]. Overall PTB was defined as delivery before 37 weeks postmenstrual gestation [24]. To create more homogenous subpopulations, outcome measures were additionally stratified by clinical presentation of PTB that were found to share common placental features [25]. Based on the findings by McElrath and colleagues [25] and to remain consistent with previous studies conducted within this cohort [22, 24], PTB was additionally classified as: (1) spontaneous PTB (defined by preterm labor or preterm premature rupture of the membranes [PPROM]; N = 49); and (2) PTB resulting from aberrant placentation or placental PTB (defined by preeclampsia or intrauterine growth restriction [IUGR]; N = 33). Deliveries for non-medical indications (i.e. prior intrauterine fetal death or prior classical cesarean section) were not analyzed separately in this study as these cases have not been found to share common underlying biological processes [25].

### Thyroid hormone measurements

Plasma samples were collected at up to four study visits: visit 1 (median 10.0 weeks; range: 4.7 to 19.1 weeks), visit 2 (median 17.9 weeks; range: 14.9 to 32.1 weeks), visit 3 (median 26.0 weeks; range: 22.9 to 36.3 weeks), and visit 4 (median 35.2 weeks; range: 33.1 to 38.3 weeks). A total of 1,443 plasma samples were assayed at the Clinical Ligand Assay Service Satellite (CLASS) Lab at University of Michigan (Ann Arbor) for TSH, total and free thyroxine (T4 and FT4, respectively), and total triiodothyronine (T3). TSH and total hormones (T3 and T4) were assayed via automated chemiluminescence immunoassay (Bayer ADVIA Centaur, Siemens Health Care Diagnostics, Inc.). FT4 was measured using direct equilibrium dialysis followed by radioimmunoassay (IVD Technologies).

The manufacturer did not provide trimester-specific reference ranges for TSH only a non-pregnant normal range of 0.35–5.50 uIU/mL. In their absence, the American Thyroid Association (ATA) recommended in 2011 the following TSH reference ranges: first trimester, 0.1–2.5 μIU/mL; second trimester, 0.2–3.0 μIU/mL; and third trimester, 0.3–3.0 μIU/mL [26]. However, the ATA has recently proposed changing the upper limit of these intervals to the non-pregnancy upper limit (~ 4.0 mU/l) [27, 28]. The FT4 pregnancy reference ranges provided by the laboratory were: first trimester, 0.7–2.0 ng/dL; second trimester, 0.5–1.6 ng/dL; and third trimester, 0.5–1.6 ng/dL. The limits of detection (LOD) were 0.01 μIU/mL for TSH, 0.1 ng/mL for T3, 0.3 μg/dL for T4, and 0.1 ng/dL for FT4. The inter-assay coefficients of variation (CV) for all hormones ranged from 2.3% (for total T3) to 10.4% (for FT4) and the intra-assay CVs ranged from 1.2% (for total T3) to 12.3% (for FT4). Thyroid hormone concentrations less than the LOD were assigned a value of LOD divided by the square root of 2 [29].

Free T3 (FT3) was not measured in this study due to sample volume constraints. Since unbound T3 is the principal bioactive hormone and potentially relevant to the mechanisms involved in PTB [30], we estimated its concentration using total T3 and the ratio of free vs. bound T4 concentrations assayed for each individual at visits 1–3. We determined the fraction of T4 that was unbound by dividing the concentration of FT4 by the concentration of T4 (both in μg/dL). Since approximately 10 times more T3 is unbound compared to T4 (~0.05% of T4 is in free form vs. ~0.5% of T3) [31], we multiplied each FT4/T4 fraction by 10 to obtain the approximate proportion of unbound T3 per sample. We then applied this proportion to the measured total T3 concentrations to estimate the fractional concentration of FT3 (in pg/mL) per sample collected from each individual at visits 1–3. While our estimations are potentially limited by differences between T3 and T4 in binding ratios of free to total hormones and in intracellular versus extracellular concentrations of unbound hormones, we used the estimated concentrations of FT3 to explore our hypothesis about its potential association with PTB and included this estimation in only the regression models assessing the odds of overall PTB.

### Statistical methods

Analyses were performed using SAS version 9.3 (SAS Institute Inc.) and R version 3.1.1 (R Foundation for Statistical Computing). The empirical histograms of total T3 and T4 approximated a normal distribution. The distributions of TSH and FT4 were right-skewed so we used the natural log transformation (ln) of these variables in statistical analyses. We used a chi-square statistic to test the differences in demographic characteristics between cases and controls.

We assessed the variability of the assayed thyroid hormones (TSH, free and total T4, and total T3) across pregnancy for the overall population as well as separately for cases and controls using several methods. First, we tabulated the median and interquartile range (25^th^ and 75^th^ percentiles) for each hormone, and evaluated differences in visits 2–4 compared to visit 1 using linear mixed models (LMMs) with a subject-specific random intercept. We also calculated the intraclass correlation coefficient (ICC) and associated 95% confidence intervals to examine the temporal variability in hormones for each subject. ICC measures the reproducibility of repeated measures from the same subject and is the ratio of between-subject variance to total variance (between- plus within-subject variance) [32]. ICC ranges from 0 to 1, with the latter indicating no within-subject variability [32].

In our third variability analysis, we examined the patterns of each hormone across pregnancy by fitting generalized additive mixed effects models (GAMM) using the R *mgcv* package. For each model, we used repeated measures of individual thyroid hormones and regressed them on a penalized spline of gestational age to assess potential nonlinear associations. We accounted for the correlation of repeated measures taken from the same subject by including subject-specific random intercepts and slopes. Predicted thyroid hormone concentrations were plotted in relation to gestational age at time of sample collection to examine patterns across pregnancy. To test whether the observed patterns varied by case-control status, we included an interaction term between PTB and gestational age.

We used logistic regression to explore associations between increases in individual thyroid hormones and the odds of PTB. Crude models included gestational age at time of sample collection. Full models were additionally adjusted for maternal age at enrollment, body mass index (BMI) at time of sample collection, parity, health insurance provider, and educational attainment. We chose age, BMI, and parity as covariates *a priori* based on their biological relevance to maternal thyroid hormone concentrations and PTB [33–37]. We identified the additional covariates based on ≥ 10% change in the main effect estimates when added to the models in a forward stepwise procedure.

We stratified logistic regression models by study visit of sample collection. We excluded data collected at visit 4 from all regression analyses in order to avoid potential bias resulting from a disproportionate number of controls compared to cases providing plasma samples at this study visit. To reveal potential biological pathways, we explored these relationships by obstetric presentation of PTB that have been previously hypothesized to share common underlying mechanisms [25]. Specifically, we repeated these stratified analyses for each subtype of PTB (spontaneous and placental PTB), adjusting logistic regression analyses for gestational age at time of sample collection, maternal age at enrollment, and maternal race. Since the gestational age ranges varied considerably for each study visit of sample collection, we also explored narrower windows of susceptibility in a separate sensitivity analysis by stratifying regression models for overall PTB by five-week intervals of gestational age (e.g., 5–10 weeks, 10–15 weeks, etc.).

## Results

The demographic characteristics of the study population by PTB status are presented in **Table 1**and are consistent with our prior publications [24]. Overall, the study population was predominately white, highly educated, and non-smokers. The majority of women were giving birth for the first time and a greater proportion of women delivering preterm were obese (>30 kg/m^2^) compared to controls (31% of cases vs. 20% of controls).

**Table 1:** Population demographic characteristics by cases (N = 116) and controls (N = 323).

Population Characteristics		Cases	Controls
		N (%)	N (%)
Age	18–24 years old	10 (9)	44 (14)
	25–29 years old	25 (22)	67 (21)
	30–34 years old	49 (42)	127 (39)
	35+ years old	32 (28)	85 (26)
Race	White	65 (56)	182 (56)
	African-American	21 (18)	54 (17)
	Other	30 (26)	87 (27)
Education	High School	21 (18)	46 (15)
	Technical School	25 (22)	51 (16)
	Junior College or some college	34 (30)	93 (30)
	College graduate	35 (30)	124 (39)
Health Insurance Provider	Private	94 (82)	250 (80)
	Public	20 (18)	63 (20)
BMI at Initial Visit	<25 kg/m^2^	51 (44)	176 (54)*
	25–30 kg/m^2^	29 (25)	84 (26)
	>30 kg/m^2^	36 (31)	63 (20)
Tobacco Use	Smoked during pregnancy	11 (9)	20 (6)
	No smoking during pregnancy	105 (91)	297 (94)
Alcohol Use	Alcohol use during pregnancy	1 (1)	12 (4)
	No alcohol use during pregnancy	113 (99)	299 (96)
Fetal sex	Male	50 (43)	148 (46)
	Female	66 (57)	175 (54)
Parity	Nulliparous	50 (43)	147 (45)
	Primiparous	32 (28)	112 (35)
	Multiparous	34 (29)	64 (20)

Abbreviations: BMI, Body Mass Index

* p<0.05 for chi-square test

### Variability in hormones across pregnancy

The distributions of thyroid hormones by study visit of sample collection are reported in **Table 2**for the overall population and by PTB status. Results from linear mixed models (LMMs) indicated that thyroid hormone concentrations varied by study visit of sample collection for both cases and controls. Intraclass correlation coefficients (ICCs) showed the lowest temporal reliability for FT4 and the highest reliability for total hormones (T3 and T4).

**Table 2:** Median (25th-75th) concentrations and intraclass correlation coefficient (ICCs) of thyroid hormone parameters by case-control status and study visit of sample collection.

Study Visit	TSH (μIU/mL)	FT4 (ng/dL)	T4 (μg/dL)	T3 (ng/mL)
**All Samples (N = 1756 observations)**				
visit 1 [ref]	0.92 (0.54, 1.50)	1.37 (1.15, 1.62)	10.1 (8.78, 11.4)	1.32 (1.13, 1.62)
visit 2	1.34 (0.97, 1.90)*	1.13 (0.90, 1.30)*	10.6 (9.60, 11.9)*	1.61 (1.35, 1.92)*
visit 3	1.28 (0.93, 1.70)*	1.00 (0.81, 1.18)*	10.4 (9.20, 11.5)*	1.66 (1.38, 1.96)*
visit 4	1.39 (0.97, 1.93)*	0.96 (0.77, 1.17)*	10.0 (9.00, 11.5)	1.66 (1.41, 2.02)*
**ICC (95%CI)**	**0.51 (0.46, 0.57)**^†^	**0.18 (0.13, 0.24)**^†^	**0.67 (0.63, 0.71)**	**0.62 (0.57, 0.67)**
**Cases (N = 116; 464 observations)**				
visit 1 [ref]	0.94 (0.51, 1.40)	1.35 (1.08, 1.55)	10.4 (8.93, 11.6)	1.38 (1.25, 1.68)
visit 2	1.24 (0.89, 1.83)*	1.13 (0.83, 1.30)*	10.6 (9.85, 12.1)*	1.70 (1.32, 2.09)*
visit 3	1.24 (0.96, 1.73)*	0.95 (0.79, 1.15)*	10.5 (9.45, 11.8)*	1.83 (1.55, 2.19)*
visit 4	1.54 (1.00, 2.02)*	0.96 (0.75, 1.24)*	10.7 (9.18, 11.9)	1.92 (1.45, 2.13)*
**ICC (95%CI)**	**0.47 (0.35, 0.58)**^†^	**0.32 (0.20, 0.43)**^†^	**0.59 (0.49, 0.68)**	**0.62 (0.51, 0.71)**
**Controls (N = 323; 1292 observations)**				
visit 1 [ref]	0.91 (0.55, 1.52)	1.39 (1.17, 1.65)	10.0 (8.70, 11.2)	1.30 (1.11, 1.60)
visit 2	1.39 (0.99, 1.91)*	1.14 (0.94, 1.30)*	10.6 (9.43, 11.8)*	1.60 (1.37, 1.87)*
visit 3	1.30 (0.90, 1.70)*	1.00 (0.82, 1.20)*	10.3 (9.10, 11.3)*	1.60 (1.35, 1.87)*
visit 4	1.34 (0.97, 1.92)*	0.96 (0.77, 1.17)*	10.0 (8.90, 11.4)	1.65 (1.39, 1.99)*
**ICC (95%CI)**	**0.53 (0.46, 0.59)**^†^	**0.15 (0.09, 0.22)**^†^	**0.69 (0.65, 0.74)**	**0.61 (0.55, 0.67)**

Abbreviations: CI, confidence interval; ICC, intraclass correlation coefficient.

* Indicates significant difference (p<0.05) in thyroid hormone concentration at the study visit compared to the reference (visit = 1) using linear mixed models with a subject-specific random intercept.

^† ^ICCs calculated using ln-transformed concentrations.

**Fig 1:**
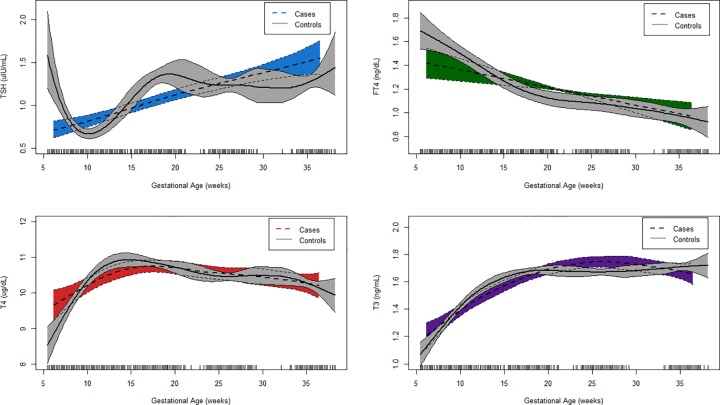
Predicted thyroid hormone concentrations across pregnancy by case-control status.

Smoothed plots of predicted thyroid hormone concentrations in association with gestational age at sample collection in cases and controls are presented in **Fig 1**. The observed trajectories of each hormone across gestation were similar to the pattern of results reported in **Table 2.** Interaction terms from generalized additive mixed effects models (GAMMs) indicated that trends in hormone concentrations across pregnancy were significantly different between cases and controls (p<0.001 for all hormones). The smoothed plot for TSH showed that concentrations were greater in controls in early pregnancy and subsequently decreased to lower concentrations than those observed in cases in the latter half of the first trimester. Predicted values of FT4 in controls were also higher in samples taken in early pregnancy, but converged to similar concentrations as those observed in cases as pregnancy progressed. Total thyroid hormone concentrations (T4 and T3) increased across gestation in both cases and controls, with a greater increase observed between approximately 5 and 15 weeks of gestation in controls.

### Gestational age-stratified analyses

Adjusted odds ratios (OR) of overall PTB in relation to a unit increase in thyroid hormone concentrations are presented in **Table 3**by study visit of sample collection. ORs from fully adjusted logistic regression models were similar to those observed in crude models. At visits 1 and 3, a one ng/dL decrease in ln-transformed FT4 was associated with approximately two times the odds of PTB. Similar to results detected for FT4, ORs for overall PTB were reduced for estimated FT3 at visits 1 and 3, although these associations were not statistically significant (data not shown).

**Table 3:** Adjusted odds ratios (95% CI) of overall preterm birth (N = 116 cases) associated with a unit increase in thyroid hormone parameters.

	Visit 1 (median 10 weeks of gestation)			Visit 2 (median 18 weeks of gestation)			Visit 3 (median 26 weeks of gestation		
Thyroid Hormone	N	OR (95%CI)	p-value	N	OR (95%CI)	p-value	N	OR (95%CI)	p-value
	(cases, controls)			(cases, controls)			(cases, controls)		
ln-TSH	81, 220	0.91 (0.71, 1.16)	0.43	86, 221	0.88 (0.61, 1.28)	0.52	75, 219	1.43 (0.85, 2.46)	0.19
ln-FT4	98, 257	0.57 (0.33, 1.00)	0.05	96, 260	0.97 (0.60, 1.54)	0.89	88, 247	0.53 (0.34, 0.84)	<0.01*
T4	100, 246	1.12 (0.99, 1.27)	0.07	92, 253	1.11 (0.96, 1.28)	0.16	85, 235	1.13 (0.99, 1.29)	0.08
T3	76, 212	2.52 (1.20, 5.31)	0.01*	82, 209	1.71 (0.81, 3.60)	0.16	70, 204	3.40 (1.56, 7.40)	<0.01*

Adjusted models include gestational age at time of sample collection, maternal age at enrollment, body mass index (BMI) at time of sample collection, parity, health insurance provider, and educational attainment.

* p<0.05

For total hormones, total T4 concentrations were suggestively associated with an increase in odds of overall PTB (p = 0.07–0.16). A unit increase of total T3 was associated with a two- to threefold increase in odds of overall PTB at all study visits with the exception of visit 2. Associations for TSH were null at all time points.

Narrower windows of gestational age were explored by stratifying ORs by five-week intervals of gestational age at time of sample collection (**S1 Table**). Results from this sensitivity analysis were similar to those reported in **Table 3**by study visit of sample collection. Significant elevated ORs were observed for total T3 measured in samples taken in early (5–10 weeks) and mid- to late pregnancy (20–25 weeks and 25–30 weeks). At these same time points, reduced albeit nonsignificant, ORs were detected for FT4.

**Fig 2:**
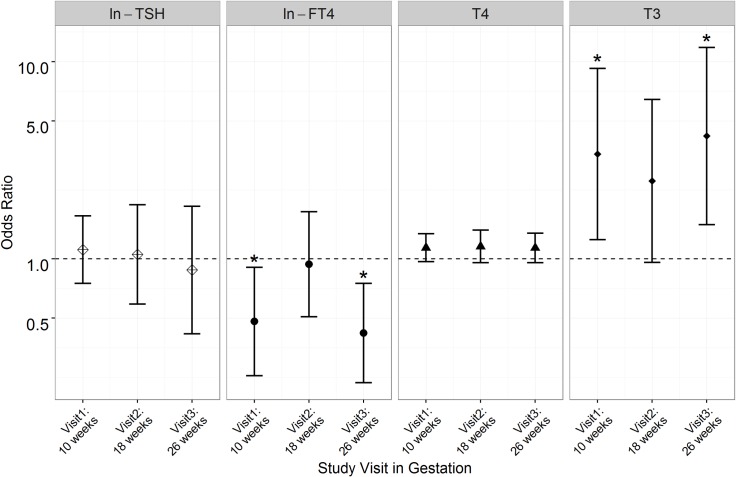
Adjusted odds ratios of spontaneous preterm birth (N = 49 cases) associated with a unit increase in thyroid hormone concentrations (*p<0.05).

**Fig 3:**
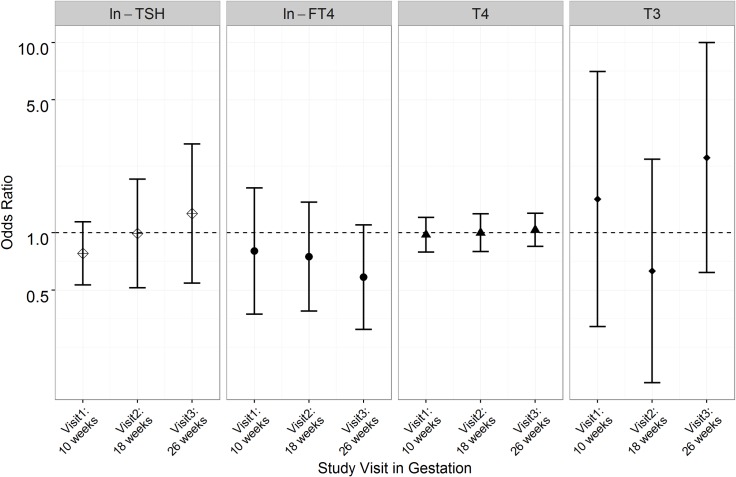
Adjusted odds ratios of placental preterm birth (N = 33 cases) associated with a unit increase in thyroid hormone concentrations (*p<0.05).

**Figs 2**and **3**show visit-specific associations between measured thyroid hormone concentrations and odds of spontaneous and placental PTB, respectively (data reported in **S2**and **S3 Tables**). For spontaneous PTB, reduced ORs for FT4 and estimated FT3 at visits 1 and 3 were similar in direction to those observed for overall PTB but were stronger and statistically significant for this subtype. The results for total T3 were in the opposite directions as those observed for FT4, and were significantly elevated at study visits 1 and 2. For placental PTB, no significant associations were observed for any of the hormones.

## Discussion

In this nested case-control study drawn from a large prospective birth cohort, we characterized the temporal patterns of thyroid function parameters across gestation. We explored windows of vulnerability for the risk of PTB using plasma samples collected at up to four time points in pregnancy. Additionally, we evaluated whether the effects of these subclinical hormonal deviations on the risk of PTB varied by clinical presentation.

### Profiles of hormones across pregnancy

This is the first study to evaluate the differences in the variability and trajectories of maternal thyroid hormone concentrations across pregnancy between women delivering preterm and at term. Various physiological changes that accompany the normal pregnancy state increase the demands of the maternal thyroid gland [4, 38]. In response to the estrogen-stimulated rise in the transport protein, thyroxine-binding globulin (TBG), there is a concomitant rise in total T3 and T4 in the first half of pregnancy until a new steady state is reached [4]. Also in the first trimester, there is a transient lowering of circulating TSH that coincides with peak human chorionic gonadotropin (hCG) concentrations [4]. Due to the structural homology between hCG and TSH molecules, hCG binds to the TSH receptor and exerts a stimulatory effect–the increased hormonal output of FT4 results in the lowering of TSH levels via the negative feedback system [4, 39]. Following the initial increase in FT4 between approximately 6 and 10 weeks of gestation as a result of the high placental production of hCG during this time period [40], FT4 subsequently decreases over pregnancy [4]. In the present study, the trajectories of thyroid hormone parameters in women delivering at term were consistent with what has been reported in the medical literature. Differences in the temporal hormonal patterns between cases and controls were most evident in the first trimester of pregnancy.

We found that total T3 and T4 concentrations were lower in controls in early pregnancy, and rose to similar or slightly greater concentrations as cases by the end of the first trimester (**Fig 1**). An upward trend in total hormones was observed in both groups until approximately 20 to 25 weeks of gestation, when a more stable concentration was reached. For TSH, concentrations were greater in controls than in cases in the earlier half of the first trimester. In controls, we observed the characteristic sharp decrease in TSH in the first trimester followed by an increase in concentrations until approximately 20 to 25 weeks of gestation (**Fig 1).** Whereas TSH concentrations fluctuated across gestation in controls and exhibited patterns similar to those reported in the literature, there was a constant upward slope in concentrations across pregnancy in women delivering preterm. However, it is uncertain whether this somewhat linear trend is a consequence of a smaller sample size in cases or if this pattern represents the true trajectory of TSH in women delivering preterm. Finally, we detected downward trends in FT4 across gestation in both cases and controls, with greater concentrations of FT4 observed in controls in early pregnancy (**Fig 1**). In response to peak hCG production in normal pregnancy, TSH decreases to its lowest concentration and FT4 to its highest concentration between approximately 9 and 12 weeks of gestation [40]. The temporal differences in the trends of TSH and FT4 between cases and controls that we observed in early pregnancy–specifically, the higher concentrations of TSH and lower concentrations of FT4 in cases compared to controls around approximately 10 weeks of gestation–may indicate a lack of thyroidal response to hCG in women delivering preterm [41]. In their recent study, Korevaar et al. observed an impaired thyroidal response to hCG in thyroperoxidase antibody (TPOAb)-positive pregnant women [41]. While TPOAb positivity is a risk factor for premature delivery [19], we did not assess thyroid autoimmunity in our study participants and therefore cannot examine the extent to which thyroid autoimmunity modifies the relationships between subclinical changes in thyroid function parameters and the risk of PTB in this study.

### Associations by gestational age

In the present study, we found that a unit decrease in FT4 was associated with an approximate twofold increase in the odds of overall PTB at median 10 weeks of gestation. These findings are in agreement with a previous study showing associations between low FT4 concentrations (<2.5^th^ percentile) at median 13 weeks of pregnancy and an increased risk of preterm delivery [19]. However, null associations have been reported by other studies for low FT4 in early pregnancy [42–45] and for continuous measures of FT4 sampled in the first half of gestation [46, 47]. Currently, there are no published data on the relationship of total T3 concentrations with PTB. However, our nonsignificant findings for estimated concentrations of FT3 and overall PTB are compatible with an earlier birth cohort study in which a lack of association was observed in early pregnancy [46].

Our null findings for TSH contrast with the results reported in studies showing associations between PTB and elevated TSH concentrations in early pregnancy [20, 48] and maternal subclinical hypothyroidism (defined as elevated TSH with normal FT4) [42, 49, 50]. However, these results were not confirmed by other studies [13, 17, 45, 46, 51].

The observed dissimilarities between our analyses and findings reported previously may be due to differences in assay methods used to measure FT4 (electrochemiluminescence immunoassay vs. direct equilibrium dialysis followed be radioimmunoassay), variability in the classification of subclinical thyroid dysfunction (e.g., differing statistical cutoff points to define elevated TSH or low FT4), inconsistent ascertainment of PTB (e.g., gestational age based on self-reported last menstrual period vs. first trimester ultrasound-validated measurements), and/or the proportion of spontaneous versus iatrogenic PTB cases. Furthermore, no other studies assessed additional time points outside of the first or second trimester.

One of the strengths of our study was our analyses by subtype of PTB. Only one previous study has examined associations between thyroid hormone concentrations and PTB with attention to presentation at delivery [52]. In that study, women with spontaneous preterm delivery (delivery <34 weeks) had significantly reduced concentrations of FT4 (within the normal range) measured in the first trimester compared to women delivering at term, although no differences were observed for TSH between the two groups. In our analysis we observed odds ratios that were greater in magnitude and more precise in models of spontaneous PTB alone, specifically for FT4 and total T3. While these findings may be due to differences in sample size between the two stratified analyses, our results for spontaneous PTB suggests that changes in these hormone concentrations during gestation may have particular consequences for spontaneous preterm labor and/or PPROM. For placental PTB, we did not observe any significant associations. Specifically, our generally null results for TSH are in contrast to studies showing associations between abnormally elevated TSH concentrations and an increased risk of preeclampsia [53] and IUGR [54], which are characteristics of placental PTB [25]. Additional studies with larger sample sizes are required to disentangle the relationships between subclinical maternal thyroid dysfunction and subtype of PTB, and to identify the underlying biological mechanisms potentially driving these associations.

It is possible that the observed fluctuations in thyroid function parameters are a result of other underlying physiological processes that ultimately lead to PTB. Spontaneous PTB is strongly associated with inflammation at the maternal-fetal interface [55, 56]. We previously demonstrated that the pro-inflammatory cytokine, interleukin-6 (IL-6), is a strong predictor of spontaneous preterm delivery in the current study population [57]. Mild thyroid hormone dysfunction at various time points in pregnancy may contribute to the inflammatory processes involved in the pathogenesis of spontaneous PTB, or vice versa. Indeed, human health studies have shown increased pro-inflammatory markers, including IL-6, in overt and subclinical hypothyroid patients [58, 59]. Consistent with this hypothesis, we found strong and highly significant inverse relationships between free hormones and spontaneous PTB in early and/or late pregnancy.

### Strengths and limitations

The primary strengths of our study was our repeated measures of thyroid function parameters collected in each trimester of pregnancy and our accurately defined clinical outcomes. Our longitudinal study design permitted an assessment of the variability in individual parameters across gestation in cases and controls as well as time points in pregnancy during which subclinical thyroidal disturbances may have a more profound effect on the risk of preterm birth. Additionally, our assay method for measuring FT4 using equilibrium dialysis is considered analytically accurate and is preferred over traditional immunoassays since measurements are not affected by thyroid hormone binding protein concentrations, which increase in pregnancy [60, 61]. Despite these strengths, our study was limited by the lack of assessment of the thyroid autoimmunity of our study participants due to biological sample volume constraints. As mentioned previously, the presence of thyroid anti-thyroid antibodies have been found to modify the relationships between circulating thyroid hormone concentrations and adverse birth outcomes [19, 26].

## Conclusions

In conclusion, our results support previous studies showing the potential for subclinical changes in thyroid hormone concentrations in pregnancy to influence the risk of PTB. Our stratified analyses showed that these effects may vary by gestational age and clinical presentation of PTB. Additional human health and animal studies should take these findings into account when trying to elucidate the mechanism(s) of subclinical thyroid dysfunction in the pathogenesis of PTB.

## Supporting Information

S1 Table Adjusted odds ratios (95% CI) of overall preterm birth (N = 116 cases) associated unit increase in thyroid hormone concentrations. (DOCX)

S2 Table Adjusted odds ratios (95% CI) of spontaneous preterm birth associated with a unit increase in thyroid hormone parameters. (DOCX)

S3 Table Adjusted odds ratios (95% CI) of placental preterm birth associated with a unit increase in thyroid hormone parameters. (DOCX)
