# Correction: Longitudinal Profiles of Thyroid Hormone Parameters in Pregnancy and Associations with Preterm Birth

**DOI:** 10.1371/journal.pone.0212950

**Published:** 2019-02-21

**Authors:** Lauren E. Johns, Kelly K. Ferguson, Thomas F. McElrath, Bhramar Mukherjee, Ellen W. Seely, John D. Meeker

In Table 1 the values for “Fetal sex” are incorrectly swapped. The first value under “Fetal sex” should be Female and the second value should be Male. Please see the corrected Table 1 here.

**Table 1:** Population demographic characteristics by cases (N = 116) and controls (N = 323).

Population Characteristics		Cases N (%)	Controls N (%)
Age	18–24 years old	10 (9)	44 (14)
	25–29 years old	25 (22)	67 (21)
	30–34 years old	49 (42)	127 (39)
	35+ years old	32 (28)	85 (26)
Race	White	65 (56)	182 (56)
	African-American	21 (18)	54 (17)
	Other	30 (26)	87 (27)
Education	High School	21 (18)	46 (15)
	Technical School	25 (22)	51 (16)
	Junior College or some college	34 (30)	93 (30)
	College graduate	35 (30)	124 (39)
Health Insurance Provider	Private	94 (82)	250 (80)
	Public	20 (18)	63 (20)
BMI at Initial Visit	<25 kg/m^2^	51 (44)	176 (54) *
	25–30 kg/m^2^	29 (25)	84 (26)
	>30 kg/m^2^	36 (31)	63 (20)
Tobacco Use	Smoked during pregnancy	11 (9)	20 (6)
	No smoking during pregnancy	105 (91)	297 (94)
Alcohol Use	Alcohol use during pregnancy	1 (1)	12 (4)
	No alcohol use during pregnancy	113 (99)	299 (96)
Fetal sex	Female	50 (43)	148 (46)
	Male	66 (57)	175 (54)
Parity	Nulliparous	50 (43)	147 (45)
	Primiparous	32 (28)	112 (35)
	Multiparous	34 (29)	64 (20)

Abbreviations: BMI, Body Mass Index

*p<0.05 for chi-square test
