# Supplementary material for: Longitudinal Profiles of Thyroid Hormone Parameters in Pregnancy and Associations with Preterm Birth
Source: PLoS One. 2017 Jan 6;12(1):e0169542. doi: 10.1371/journal.pone.0169542 (PMC5217954; doi:10.1371/journal.pone.0169542)
Supplement: S1 Table — (DOCX) [file pone.0169542.s001.docx]

| **S1 Table. Adjusted odds ratios (95% CI) of overall preterm birth (N=116 cases) associated unit increase in thyroid hormone concentrations.** | | | | | | | | | | | | | | | | | |  |
| --- | --- | --- | --- | --- | --- | --- | --- | --- | --- | --- | --- | --- | --- | --- | --- | --- | --- | --- |
| Gestational Age (weeks) |  | ln-TSH | |  |  | ln-FT4 | |  | T4 | |  | | T3 | | | | | |
|  | N (cases, controls) | OR (95%CI) | p-value |  | N (cases, controls) | OR (95%CI) | p-value |  | N (cases, controls) | OR (95%CI) | p-value |  | | N (cases, controls) | OR (95%CI) | p-value |  |  |
| 5-10 | 44, 122 | 0.74 (0.52, 1.07) | 0.11 |  | 53, 143 | 0.44 (0.18, 1.11) | 0.08 |  | 54, 137 | 1.12 (0.93, 1.35) | 0.22 |  | | 42, 118 | **3.05 (0.98, 9.50)** | **0.05** |  |  |
| 10-15 | 36, 98 | 1.18 (0.77, 1.81) | 0.46 |  | 44, 114 | 0.63 (0.29, 1.34) | 0.23 |  | 45, 109 | 1.11 (0.93, 1.34) | 0.25 |  | | 34, 95 | 2.52 (0.86, 7.39) | 0.09 |  |  |
| 15-20 | 84, 211 | 0.83 (0.58, 1.17) | 0.29 |  | 94, 249 | 0.98 (0.60, 1.62) | 0.95 |  | 90, 243 | 1.14 (0.98, 1.31) | 0.09 |  | | 79, 201 | 1.80 (0.83, 3.88) | 0.14 |  |  |
| 20-25 | 31, 48 | 1.46 (0.49, 4.34) | 0.49 |  | 33, 58 | 0.44 (0.18, 1.05) | 0.06 |  | 32, 52 | 1.11 (0.82, 1.50) | 0.49 |  | | 28, 46 | **4.52 (1.05, 19.4)** | **0.04** |  |  |
| 25-30 | 47, 178 | 1.49 (0.77, 2.89) | 0.23 |  | 58, 197 | 0.61 (0.36, 1.01) | 0.06 |  | 56, 190 | 1.07 (0.92, 1.25) | 0.38 |  | | 45, 164 | **2.59 (1.03, 6.51)** | **0.04** |  |  |
| Adjusted models include gestational age at time of sample collection, maternal age at enrollment, body mass index (BMI) at enrollment, parity, health insurance provider, and educational attainment. | | | | | | | | | | | | | | | | | |  |
