# Supplementary material for: Longitudinal Profiles of Thyroid Hormone Parameters in Pregnancy and Associations with Preterm Birth
Source: PLoS One. 2017 Jan 6;12(1):e0169542. doi: 10.1371/journal.pone.0169542 (PMC5217954; doi:10.1371/journal.pone.0169542)
Supplement: S2 Table — (DOCX) [file pone.0169542.s002.docx]

| **S2 Table. Adjusted odds ratios (95% CI) of spontaneous preterm birth associated with a unit increase in thyroid hormone parameters.** | | | | | | | | | | | |
| --- | --- | --- | --- | --- | --- | --- | --- | --- | --- | --- | --- |
| Thyroid  Hormone | Visit 1  (median 10 weeks of gestation) | | |  | Visit 2  (median 18 weeks of gestation) | | |  | Visit 3  (median 26 weeks of gestation) | | |
|  | N | OR (95% CI) | p-value |  | N | OR (95% CI) | p-value |  | N | OR (95% CI) | p-value |
|  | (cases, controls) |  |  |  | (cases, controls) |  |  |  | (cases, controls) |  |  |
| ln-TSH | 36, 233 | 1.11 (0.75, 1.65) | 0.60 |  | 38, 229 | 1.05 (0.59, 1.88) | 0.86 |  | 32, 228 | 0.88 (0.42, 1.85) | 0.73 |
| ln-FT4 | 42, 272 | **0.48 (0.25, 0.91)** | **0.02** |  | 45, 269 | 0.94 (0.51, 1.74) | 0.84 |  | 38, 257 | **0.42 (0.24, 0.75)** | **<0.01** |
| T4 | 43, 260 | 1.14 (0.97, 1.34) | 0.12 |  | 42, 262 | 1.16 (0.96, 1.40) | 0.14 |  | 36, 235 | 1.14 (0.96, 1.35) | 0.15 |
| T3 | 34, 225 | **3.40 (1.25, 9.25)** | **0.02** |  | 36, 217 | 2.48 (0.96, 6.42) | 0.06 |  | 29, 213 | **4.20 (1.49, 11.8)** | **<0.01** |
| Logistic regression models adjusted for gestational age at time of sample collection, maternal age at enrollment, and maternal race. | | | | | | | | | | | |
