# Supplementary material for: Longitudinal Profiles of Thyroid Hormone Parameters in Pregnancy and Associations with Preterm Birth
Source: PLoS One. 2017 Jan 6;12(1):e0169542. doi: 10.1371/journal.pone.0169542 (PMC5217954; doi:10.1371/journal.pone.0169542)
Supplement: S3 Table — (DOCX) [file pone.0169542.s003.docx]

| **S3 Table. Adjusted odds ratios (95% CI) of placental preterm birth associated with a unit increase in thyroid hormone parameters.** | | | | | | | | | | | |
| --- | --- | --- | --- | --- | --- | --- | --- | --- | --- | --- | --- |
| Thyroid  Hormone | Visit 1  (median 10 weeks of gestation) | | |  | Visit 2  (median 18 weeks of gestation) | | |  | Visit 3  (median 26 weeks of gestation) | | |
|  | N | OR (95%CI) | p-value |  | N | OR (95%CI) | p-value |  | N | OR (95%CI) | p-value |
|  | (cases, controls) |  |  |  | (cases, controls) |  |  |  | (cases, controls) |  |  |
| ln-TSH | 21, 233 | 0.78 (0.53, 1.14) | 0.20 |  | 27, 229 | 0.99 (0.51, 1.91) | 0.98 |  | 24, 228 | 1.26 (0.54, 2.93) | 0.59 |
| ln-FT4 | 29, 272 | 0.80 (0.37, 1.72) | 0.57 |  | 29, 269 | 0.75 (0.39, 1.45) | 0.39 |  | 29, 257 | 0.58 (0.31, 1.10) | 0.10 |
| T4 | 29, 260 | 0.98 (0.79, 1.20) | 0.82 |  | 28, 262 | 1.00 (0.80, 1.26) | 1.00 |  | 26, 244 | 1.04 (0.85, 1.26) | 0.73 |
| T3 | 18, 225 | 1.50 (0.32, 7.03) | 0.60 |  | 25, 217 | 0.63 (0.16, 2.44) | 0.50 |  | 23, 213 | 2.48 (0.62, 9.90) | 0.20 |
| Logistic regression models adjusted for gestational age at time of sample collection, maternal age at enrollment, and maternal race. | | | | | | | | | | | |
